# Supplementary material for: The Gfr Uptake System Provides a Context‐Dependent Fitness Advantage to Salmonella Typhimurium SL1344 During the Initial Gut Colonization Phase
Source: Mol Microbiol. 2025 Oct 13;124(6):507–20. doi: 10.1111/mmi.70027 (PMC12675984; doi:10.1111/mmi.70027)
Supplement: Supplementary file 1 — Figures S1–S4: mmi70027‐sup‐0001‐FiguresS1‐S4.pdf. [file MMI-124-507-s003.pdf]

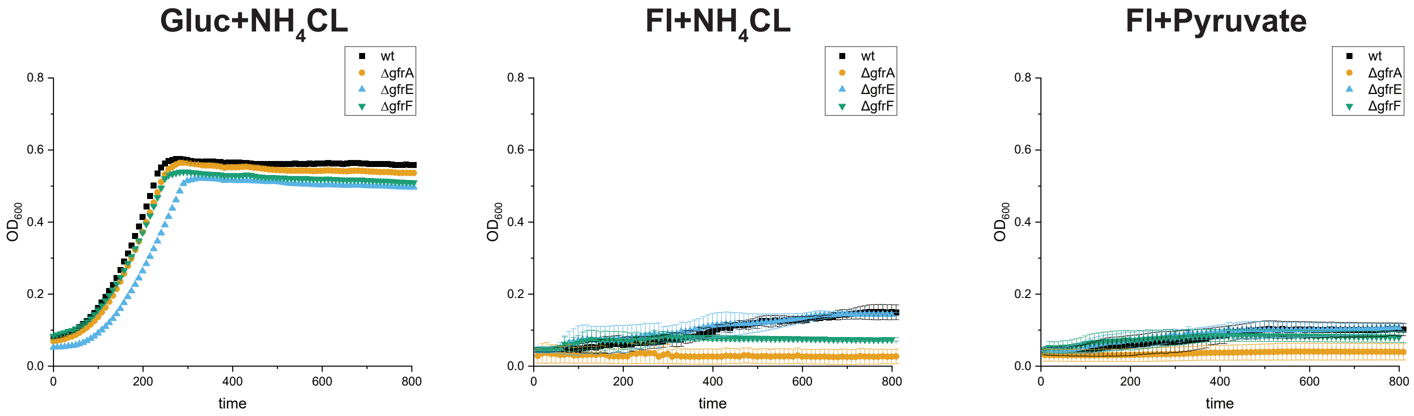

Supplemental Figure 1. Growth curves of relevant strains, presented as supplemental data for Figure 4A.

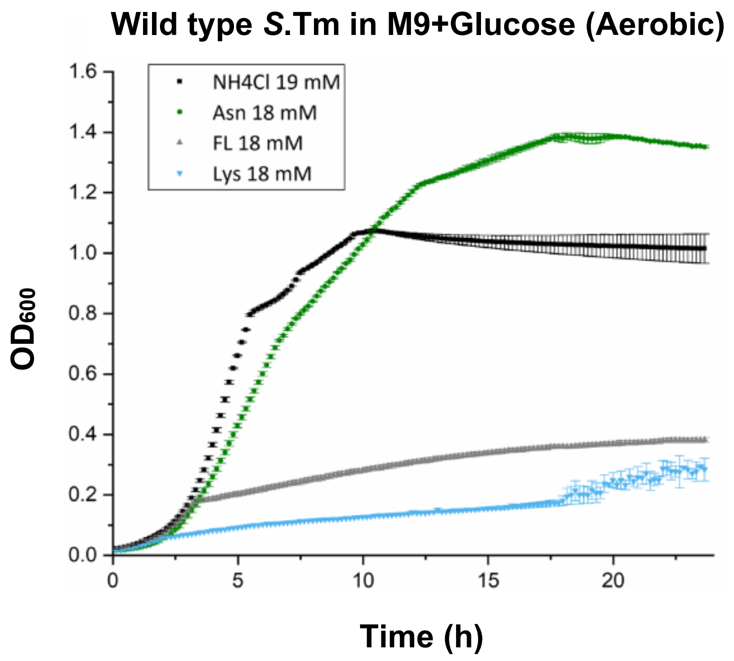

Supplementary Figure 2. Wild type *S*. Tm growth under aerobic conditions in M9 medium supplemented with 22 mM glucose with the indicated nitrogen sources (N = 3).

A

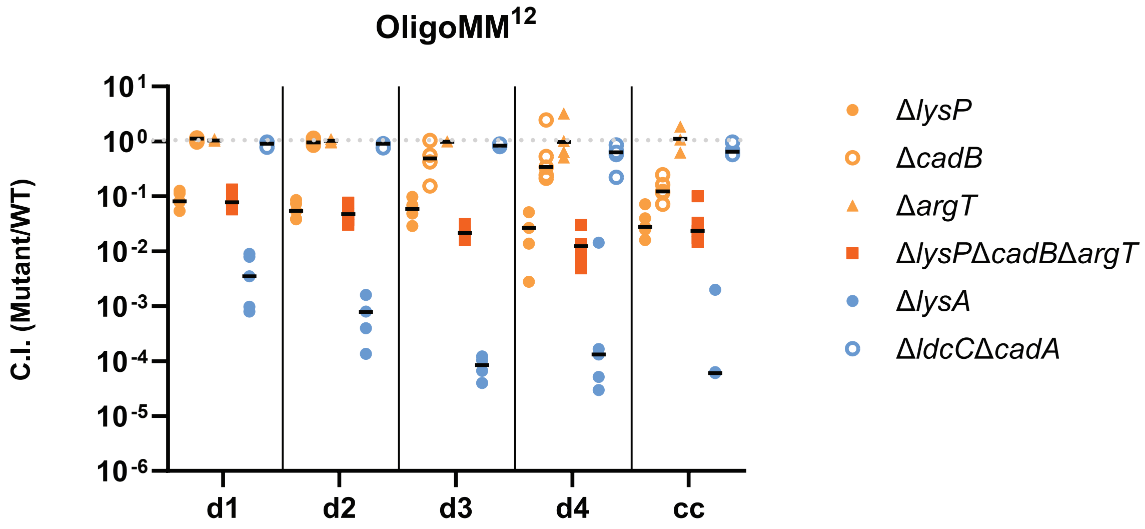

B

Competitive Indices (CI)

	ΔlysP	*ΔcadB*	*ΔargT*	*ΔldcC ΔcadA*	*ΔlysA*	*ΔlysP ΔcadB ΔargT*	*ΔlysP ΔcadB ΔargT Δgfr*
d1	0.081557301	1.12924919	1.04007881	0.911231661	0.003497795	0.077657022	0.026246096
d2	0.054241698	0.959444339	1.024750004	0.911597677	0.00079051	0.047576598	0.015112599
d3	0.058960204	0.489387965	0.991487996	0.843407913	8.50406E-05	0.021495358	0.01550965
d4	0.026720329	0.342205445	0.974712563	0.636404315	0.000132291	0.012409775	0.005274851
cc	0.027577911	0.122344867	1.111772102	0.647586247	6.06331E-05	0.023594385	0.010717381

Supplemental Figure 3. Competitive index (CI) data corresponding to Figure 5C and 5D. (A) Plotted CI values supplementing the analysis shown in Figure 5C. (B) Individual CI values corresponding to Figures 5C and 5D.

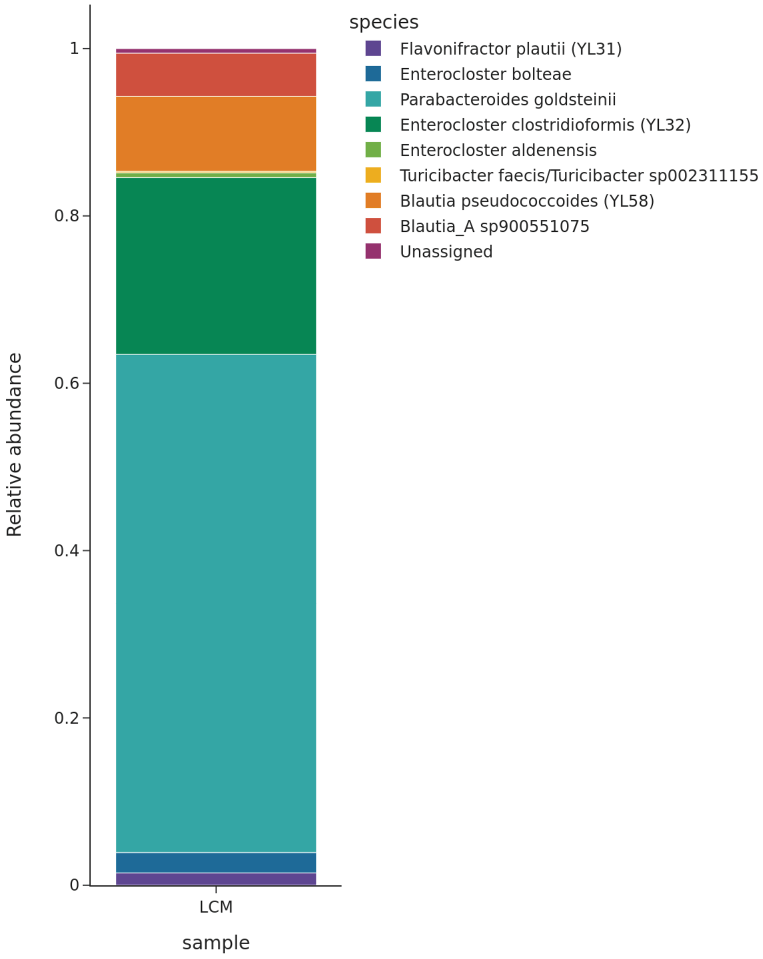

Supplemental Figure 4. Taxonomic composition of LCM. Relative abundance of microbial taxa in cecum content of LCM mice as determined by mOTU profiling.
